# Biomechanics and functional morphology of a climbing monocot

**DOI:** 10.1093/aobpla/plw005

**Published:** 2016-01-27

**Authors:** Linnea Hesse, Sarah T. Wagner, Christoph Neinhuis

**Affiliations:** 1 Institut für Botanik, Technische Universität Dresden, D-01062 Dresden, Germany; 2 Institut für Spezielle Botanik, Johannes Gutenberg—Universität Mainz, D-55112 Mainz, Germany; 3 Present address: Plant Biomechanics Group Freiburg, University of Freiburg, Schänzlestrasse 1, D-79104 Freiburg im Breisgau, Germany

**Keywords:** Biomechanics, climbing plants, *Flagellaria indica*, functional morphology, monocotyledons, structural bending modulus, structural modulus of torsion, three-point bending, twist-to-bend ratio

## Abstract

Plants with a climbing growth habit possess unique biomechanical properties arising from adaptations to changing loading conditions connected with close attachment to mechanical supports. In monocot climbers, mechanical adaptation is restricted by the absence of a bifacial vascular cambium. *Flagellaria indica* was used to investigate the mechanical properties and adaptations of a monocot climber that, uniquely, attaches to the surrounding vegetation via leaf tendrils. Biomechanical methods such as three-point bending and torsion tests were used together with anatomical studies on tissue development, modification and distribution. In general, the torsional modulus was lower than the bending modulus; hence, torsional stiffness was less than flexural stiffness. Basal parts of mature stems showed the greatest stiffness while that of more apical stem segments levelled off. Mechanical properties were modulated via tissue maturation processes mainly affecting the peripheral region of the stem. Peripheral vascular bundles showed a reduction in the amount of conducting tissue while the proportion and density of the bundle sheath increased. Furthermore, adjacent bundle sheaths merged resulting in a dense ring of fibrous tissue. Although *F. indica* lacks secondary cambial growth, the climbing habit is facilitated by a complex interaction of tissue maturation and attachment.

## Abstract (precis)

Climbing monocots can develop into large bodied plants despite being confined by primary growth. In our study on *Flagellaria indica* we measured surprisingly high stem biomechanical properties (in bending and torsion) and we show that the lack of secondary growth is overcome by a combination of tissue maturation processes and attachment mode. This leads to higher densities of mechanically relevant tissues in the periphery of the stem and to the transition from self-supporting to climbing growth. The development of specialised attachment structures has probably underpinned the evolution of numerous other large bodied climbing monocot taxa.

## Introduction

One potential cause of species richness in angiosperms is a high diversity in growth form that enables colonization of a wide range of contrasting habitats (Ricklefs and Renner 1994; Bresinsky *et al*. 2008; Wagner *et al*. 2012). The development of secondary growth via cambial activity and the linked ability to change structural and mechanical properties during ontogeny are considered to be important novel properties promoting this diversity. In climbing plants, developmental changes in stem biomechanics become apparent in the transition from their juvenile self-supporting stage, which enables plants to reach and attach to host supports, to their non-self-supporting climbing stage, which tolerates mechanical stresses (Hoffmann *et al*. 2003; Rowe *et al*. 2004; Isnard and Silk 2009; Chen *et al*. 2014). During the initial self-supporting phase, stem stiffness is often achieved by a ring of primary fibres in the peripheral regions of the stem or by the early production of a dense secondary xylem (Isnard and Silk 2009). With the onset of the climbing phase, however, cambial activity often leads to a highly compliant ‘lianoid’ stem. In addition, cambial variants such as irregular, supernumerary, unilateral, concentric, multiple and lobed cambia can lead to anatomically anomalous structures in lianas (Carlquist 1988; Rowe *et al*. 2004; Isnard and Silk 2009), which contribute to their particular biomechanical properties. Thus, it is mainly cambial activity that contributes to the anatomical adaptations and modifications leading to the specific mechanical and architectural properties of climbing plants.

Therefore, the question arises as to how monocots that have lost the secondary vascular cambium realize the specific mechanical requirements of the climbing growth. These plants are limited in modulating material properties by secondarily produced tissues and thereby in their ability to generate different growth habits. This is because their growth and development are confined to primary-type growth. Nevertheless, monocots have evolved tall self-supporting tree-like forms and even climbers (Rowe *et al*. 2004, 2006; Isnard *et al*. 2005; Isnard and Silk 2009).

Compared with biomechanical studies in other angiosperm taxa, monocots are poorly investigated with respect to the biomechanics of growth forms. Studies have mainly focussed on climbing palms such as *Calamus* (rattans) and *Desmoncus*, which cling to the surrounding vegetation via specialized spines or hooks along their elongated leaf rachis (Isnard *et al*. 2005; Isnard and Silk 2009; Isnard and Rowe 2008*b*; Rowe and Isnard 2009). These studies provide valuable results on the mechanisms that monocots have developed to realize a non-self-supporting growth habit that depends on a mechanically supporting host.

*Flagellaria indica* promises further insights into biomechanical traits of monocotyledonous climbers and their mechanical adaptation due to a different attachment mechanism compared with *Desmoncus* and *Calamus. Flagellaria indica* climbs via coiled tendrils terminating the leaves and assuring a close contact to supports (Tomlinson and Posluszny 1977; Takhtadzhi⌢an 1997; Kubitzki *et al*. 1998).

*Flagellaria* is the sole genus of the family Flagellariaceae and consists of five species, which are distributed throughout the tropical and subtropical regions of the Old World (southeast Asia, tropical Africa, northern Australia and the Pacific Islands) (Kubitzki *et al*. 1998; Rabenantoandro *et al*. 2007; Wepfer and Linder 2014). *Flagellaria* species occur mainly in coastal regions, in moist lowland forests and mangroves, often along forest margins and in secondary forests (Larsen 1972; Linder 1987; Kubitzki *et al*. 1998). The solid, cane-like, aerial stems, which originate from sympodial rhizomes, can reach a length of 20 m and lack axillary buds (Kubitzki *et al*. 1998; Wepfer and Linder 2014). Dichotomizing apices have previously drawn attention to this species (Tomlinson and Posluszny 1977). Leaves are distichous with a closed leaf sheath and differentiate into coiled tendrils, which enable attachment to surrounding vegetation.

This study addresses (i) the overall biomechanical architecture of *F. indica*, (ii) the functional traits of *F. indica* by discussing the results of biomechanical measurements via bending and torsion tests in relation to anatomical observations and (iii) the mechanical adaptation of *F. indica* and other climbing monocots, despite the lack of cambial growth.

## Methods

### Sampling

**Figure 1.**
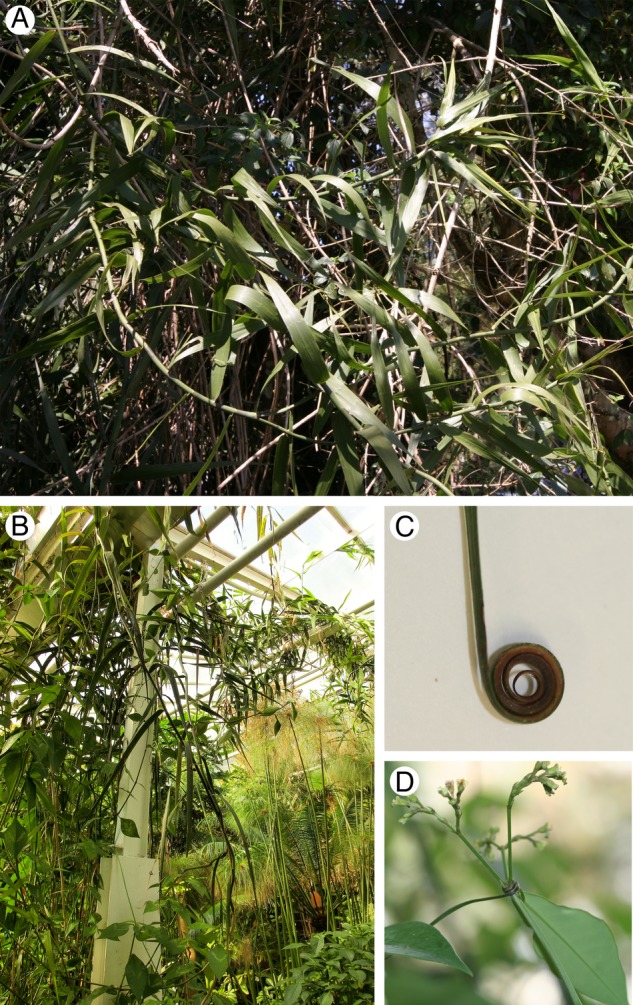
Growth habit and morphology of *F. indica*. (A) The morphology and growth habit of an individual observed in its natural habitat. (B) Morphology and growth habit of the cultivated plant. Basal self-supporting region with vertical growth; distal, climbing regions are partly fixed underneath the roof of the greenhouse. (C) The leaf apex, curled to a tendril. The adaxial side of the tendril is thickened. (D) Attachment via leaf tendril to an adjacent host support.

All investigations were carried out on one mature, intensely sprouting individual plant cultivated in a greenhouse of the Botanic Garden at Dresden, Germany (Fig. 1B). Entire aerial stems were cut for biomechanical and anatomical investigation, and were kept under moist conditions prior to measurements (maximum 24 h) to prevent dehydration and to maintain natural material properties. The longest aerial stems investigated had a length of 15 m, comparable with the stem length (15–20 m) recorded at natural sides (Larsen 1972; Kubitzki *et al*. 1998; Wepfer and Linder 2014). Branches overgrew the surrounding vegetation reaching the greenhouse roof top (5 m). Thus, growth habit resembles natural observations (Fig. 1), even though long aerial shoots were cut back by the garden staff to reduce their extensive growth. As most plant species show variation in their growth habit, also under natural conditions, the cultivated individual might also differ slightly in size, in growth rate and biomechanical traits compared with traits of other individuals in greenhouses and in the natural habitat. However, each individual, whether cultivated or growing in the wild, must cope with habitat-specific challenges, by adjustments to growth habit and architecture.

### Biomechanics

Stem segments of five aerial shoots (AS 1–5) were measured first in bending and subsequently in torsion. Aerial shoots 1–3 represented young and relatively short stems (max. 4 m), while AS 4 and 5 are older and have already reached the greenhouse roof top (max. stem length 15 m). Their apices were no longer active. The position of each tested sample was recorded to note its distance from the base (i.e. from basal to the most aerial part) and/or apex of the stem. Only relatively straight stem segments were used.

Samples were assigned to three developmental stages, based on comparable characteristics and distance from either the apex or base of the stem. Stage G1 (*n*= 6) includes most apical stem sections located in 0–1 m distance to an active apex (only AS 1–3). Stage G2 (*n*= 19) consists of samples located 4–13 m from the base (only AS 4 and 5). Stage G3 (*n*= 9) contains old, mostly basal stem sections with a maximal distance to the base of 1 m (only AS 2–5). Some additional segments of the transition zones between the defined developmental stages were also included in the measurements.

To avoid bias to measurements caused by shear forces, a suitable span-to-depth ratio of >20 in bending as well as torsion tests was maintained for all samples (Vincent 1992; Beismann *et al*. 2000; Gallenmüller *et al*. 2001). Here, span describes the distance between the two support points of the bending device or the two chucks of the torsion device, respectively; depth representing the average diameter of the sample based on three measurements along the stem segment. The diameter considers the leaf sheath, and thus, measurements of stem biomechanics reflect the entire axis.

The structural Young's (structural bending modulus) and structural torsional modulus were used to compare the developmental stages in terms of their biomechanical properties. They were calculated from flexural and torsional stiffness determined in experiments described below. The term ‘structural’ emphasizes that, unlike according to the definition of Young's modulus and the modulus of torsion, not a single material but a composite is measured. As plant stems and many other biological materials have a complex structure, both moduli are necessarily interpreted as spatially averaged values across the entire heterogeneous plant tissues (Rowe and Speck 1998; Isnard *et al*. 2005; Rowe *et al*. 2006).

Three-point-bending measurements were conducted using a Zwick/Roell BZ 2.5/TS1S bench-top mechanical testing device (Zwick/Roell, Ulm, Germany), following the protocol of Wagner *et al*. (2012). Structural Young's modulus (*E*) (referred to as bending modulus in the following) of each stem segment was determined by dividing the flexural stiffness (*EI*) by the second moment of area (*I*).

**Figure 2.**
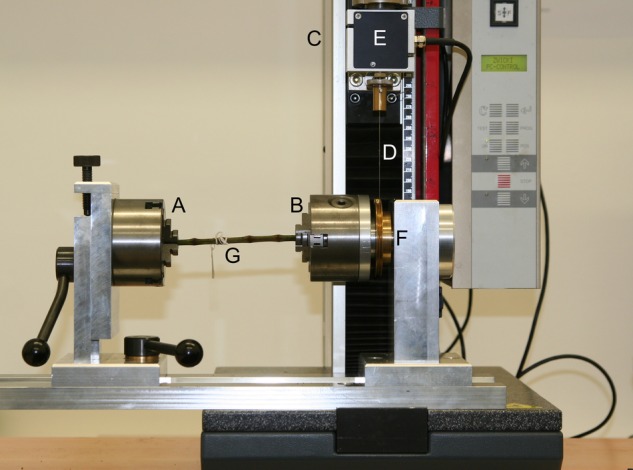
Experimental set-up for torsional loading of plant material. Tests in torsion were carried out using the Zwick/Roell testing machine. Each sample (G) is fixed to two chucks of which one is fixed (A) and the other one is rotatable (B). The moment of force is applied by moving the cross head upward (C). This movement is transmitted to the rotatable chuck by a Kevlar cord (D), which is fixed to the force transducer at the cross head (E) and to a transmission disk (F) at the rotatable chuck.

Tests in torsion were carried out using the Zwick/Roell testing machine with a set-up consisting of two chucks, one fixed (Fig. 2A) and one rotatable (Fig. 2B). The moment of force was applied by moving the cross head upward (5 mm s^−1^; Fig. 2C). This movement was transmitted to the rotatable chuck by a Kevlar cord (Grebenstein Kevlarvorfach, 15 kg lifting capacity; Fig. 2D), fixed to the force transducer at the cross head (Fig. 2E) and to a transmission disk (Fig. 2F) at the rotatable chuck. Kevlar chord was chosen as it has a low strain of rupture (2.99 %), a high tensile strength (*F*_max_ = 150 kg m s^−2^) and little internal strain, which allows measurements of both stiff and soft materials.

Each sample (Fig. 2G) was fixed to the chucks and twisted by the applied moment of force. The translation of the cross head, moving a distance *x*, was converted to an angle of torsion (φ) with the formula φ = *x* × *r*^−1^ [rad], where *r* is the radius of the transmission disk.

The modulus of torsion was calculated using the length of a sample *L* (the distance between the two chucks), the torsional constant *J*, the angle of torsion φ and the applied moment of force *T* (Niklas 1991; Etnier 2003; Vogel 2007): *G* = (*F* × *x*^−1^) × ((*r*^2^ × *L*) × *J*^−1^), in which *F* × *x*^−1^ is the slope of the linear regression of the force–deflection curve and *r* is the radius of the transmission disk.

The torsional constant *J* takes the geometry of the sample into account and thus the influence of its cross-sectional shape under torsion. The formula *J* = (*π* × 16)^−1^ × ((*a*^3^ × *b*^3^) × (*a*^2^ + *b*^2^))^−1^ for elliptic cross-sections incorporates natural variations of stem shape, with *a* being the major and *b* the minor diameter of the stem.

Torsional stiffness *GJ* is analogous to the flexural stiffness *EI*, the product of the material property *G* of the sample and its geometry factor *J*. It represents the resistance of a stem fragment to twisting loads (Vogel 2007).

The ratio of flexural stiffness *EI* and torsional stiffness *GJ* gives a dimensionless value that allows comparison of different developmental stages while considering geometrical differences. Two stem fragments can have the same *EI*/*GJ* ratio though showing differences in either material or geometrical properties or even both (Niklas 1992; Etnier 2003). Furthermore, the *EI*/*GJ* ratio allows comparing different species and provides information about the degree of anisotropy (Vogel 1992, 2007). An isotropic material would have an *EI*/*GJ* ratio of 1. A high *EI*/*GJ* ratio indicates a strong directional dependence of mechanical traits and thereby a strong anisotropy. Determining the anisotropy of the tested samples will, therefore, provide a better understanding of how the material properties are directionally dependant.

Due to the lack of secondary cambial growth, stems of *F. indica* are relatively homogenous in diameter from base to apex compared with dicotyledons. Thus, stem geometrical traits of *F. indica* cannot be as easily correlated with stem developmental traits, as it has been done in studies on non-monocots (Gallenmüller *et al*. 2001; Isnard *et al*. 2003*a*, *b*; Wagner *et al*. 2012, 2014). Instead, considering the base (here: the position where the plant emerges from the soil) and/or the apex as reference points gives insights into changes of mechanical properties in relations to developmental change.

### Statistical analysis

Material properties of the three developmental stages were statistically analysed using Kruskal–Wallis tests and pairwise Mann–Whitney *U*-tests (*post hoc* testing). The *P*-values were adjusted using Holm-correction. All statistical tests were carried out using the stats package of the freeware ‘R’ (R Development Core Team 2014). The boxplots were created using the ggplot2 package (Wickham 2009).

### Anatomy

Anatomical investigations were carried out subsequent to the biomechanical measurements to correlate tissue distribution and differentiation with biomechanical properties. Samples were preserved in 70 % ethanol, and transverse and longitudinal sections of different developmental stages were prepared with a vibratome (Microtome Hyrax V50, Carl Zeiss MicroImaging GmbH, Jena, Germany). Amplitude, frequency and razor blade speed were adjusted for each sample. Stiff samples were cut with a standard sliding wood microtome or cut by hand with a razor blade. Fully developed, rather stiff stem regions were softened with ethylenediamine solution (6.8–10 %) for 4 days prior to sectioning (Kukachka 1977; Carlquist 1982; Angyalossy *et al*. 2010). All sections were stained with safranin/astra blue and photographed using the ProgRes^®^ Capture Pro 2.7 Camera (Jenoptik) and the software Image-Pro Plus 7.0. Phloroglucinol–HCl was used to confirm the staining results by staining lignified tissues pink/red. Further anatomical investigations were conducted using a scanning electron microscope (Supra 40 VP, Zeiss) to gain better insights on tissue development and distribution. Samples were then photographed using the Software SmartSEM^®^ control.

## Results

### Growth habit of *F. indica*

**Figure 3.**
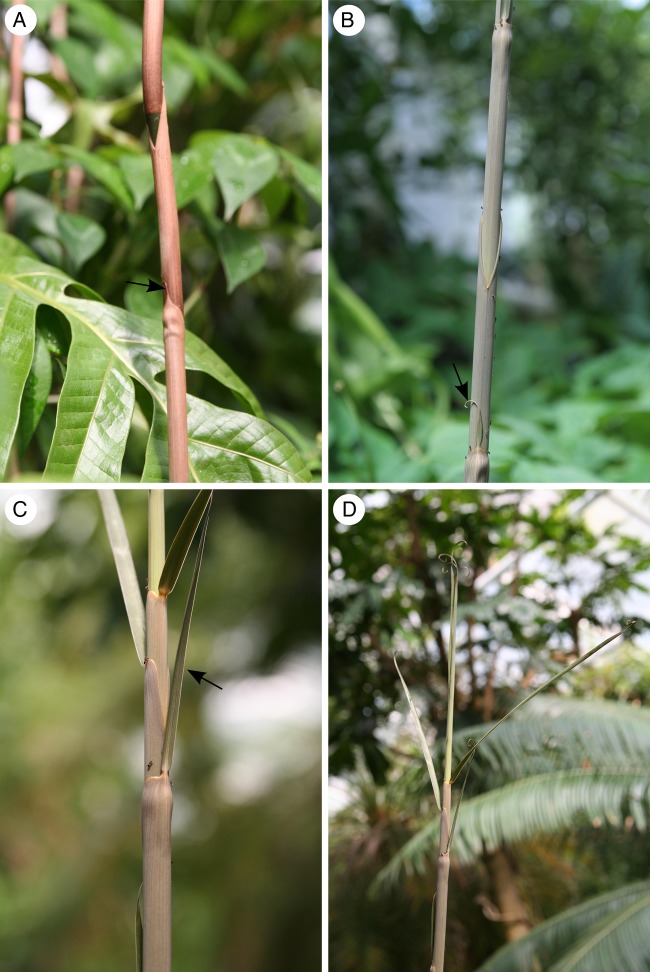
The stem surface of three developmental stages G1–G3. The black arrows point towards the lamina of the leaf. (A) Old, basal stem surface (G3) with incompletely developed (rudimentary lamina without climbing function), quickly senescing and scarious leaves, consisting mainly of sheath. (B) Leaf tendrils begin to develop at a height of 2.5–3 m, which enables the attachment to supports (basal G2 stems). (C) After a height of 2.5 m, leaves are fully developed, becoming apparent by a well-developed lamina and leaf tendrils. (D) The leaf tips begin to coil very early in their development, which allows a first attachment to the surrounding vegetation (G1).

Aerial shoots of *F. indica* show a self-supporting phase up to 2–3 m and grow vertically towards the greenhouse rooftop searching for supporting vegetation (Figs 1B and 3D). The basal regions of the stems are characterized by incompletely developed, quickly senescing and scarious leaves, consisting mainly of sheaths (Fig. 3A). They have only a rudimentary lamina and no climbing function. Leaves are fully developed at a height of 2.5 m with a well-developed lamina and leaf tendrils (Figs 1C and D and 3B and C) and extensions of the midrib, which enable attachment to supports.

Leaf tips begin to coil very early in their development (Fig. 3D) and the coiled structures allow a first attachment to the surrounding vegetation. Once support is located, tendrils begin to coil further and thicken adaxially (S. Wagner, A. Rjosk and C. Neinhuis, unpubl. data). Hence, a strong and close contact to a support is achieved (Fig. 1D). A close contact to supporting hosts could only be detected after a height of 3 m or higher was accomplished. Attachment to adjacent plants allows *F. indica* to maintain a more-or-less upright growth without relying on its own stem as mechanical support (Fig. 1A and B).

The morphology and growth habit of the cultivated plant resemble that of individuals observed in their natural habitat (e.g. Linder 1987; Kubitzki *et al*. 1998; Wepfer and Linder 2014; C. Neinhuis, pers. comm.; Fig. 1A). Nevertheless, all data obtained from greenhouse material will be discussed with caution since it may differ somewhat from that growing in natural environments.

### Biomechanical properties in bending and torsion

**Figure 4.**
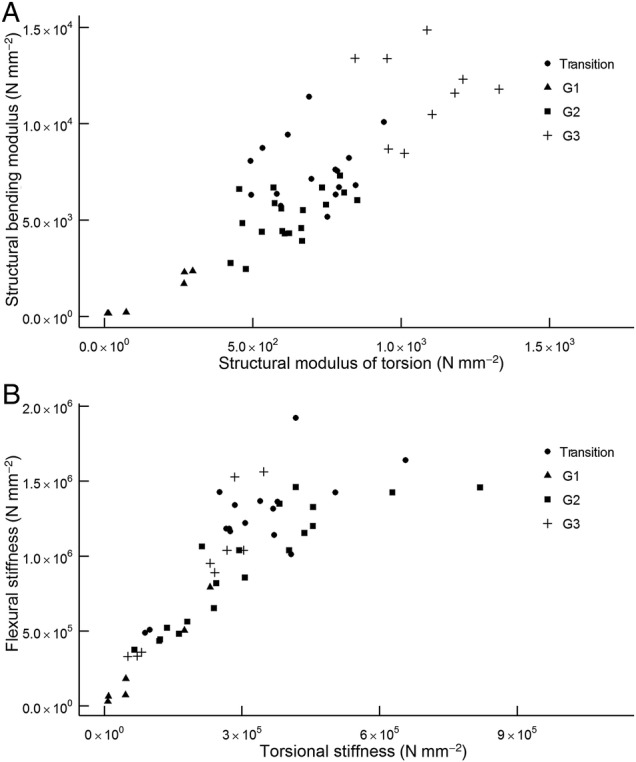
Correlation of structural bending modulus and structural modulus of torsion (A) and bending stiffness and torsional stiffness (B) of stem segments assigned to three developmental stages. Samples, which are not assigned to developmental stages, are transitional stem segments between the groups G1–G3. (A) Structural bending modulus and modulus of torsion show a linear relationship; if *E* increases, so does *G. E* is always higher than *G* (*E*> *G*). (B) Flexural stiffness always shows higher values compared with torsional stiffness (*EI*> *GJ*). If *EI* increases, so does *GJ*. Again, a linear relationship is apparent.

In general, the modulus of torsion of a stem segment is lower than its bending modulus and, if *E* increases, so does *G* (Fig. 4A). This is also the case for the flexural and torsional stiffness (Fig. 4B). Each developmental stage is characterized by particular biomechanical properties.

#### Stage G1

**Figure 5.**
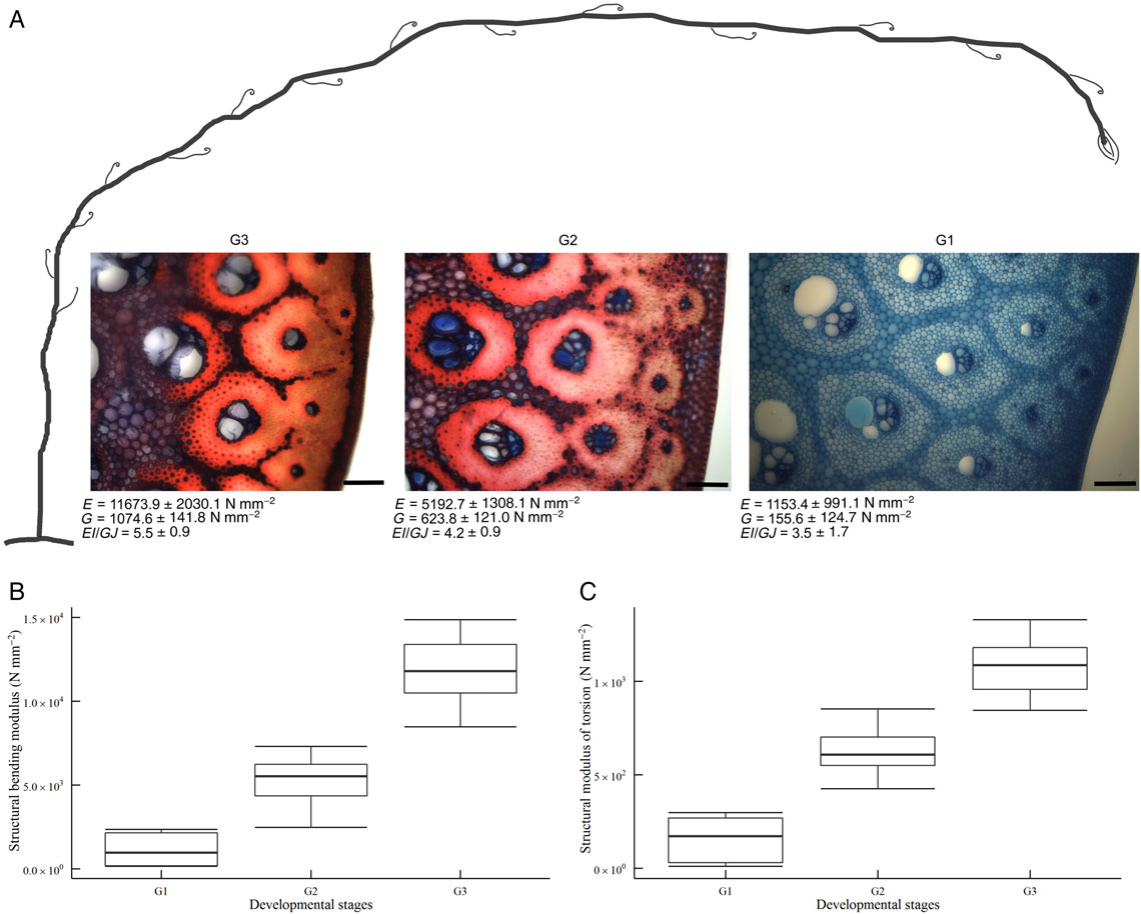
Changes of material properties during development. (A) Schematic representation of one aerial shoot. The anatomical changes during development are visualized for G1–G3 via small representative images of the peripheral region of the transverse stem section. The changes of material properties, structural bending modulus (*E*) and structural modulus of torsion (*G*), and changes of the *EI*/*GJ* ratio are also given with the mean values for each developmental stage G1–G3. (B) Boxplot showing the highly significant differences of the structural bending modulus of G1–G3. (C) Boxplot showing the highly significant differences of the structural modulus of torsion of G1–G3.

Young apical stem regions show the lowest values with moduli of torsion below 200 N mm^−2^ (155.6 ± 124.7 N mm^−2^) and bending moduli below 2000 N mm^−2^ (1153.4 ± 991.1 N mm^−2^) (G1 in Figs 4A and 5).

#### Stage G2

**Figure 6.**
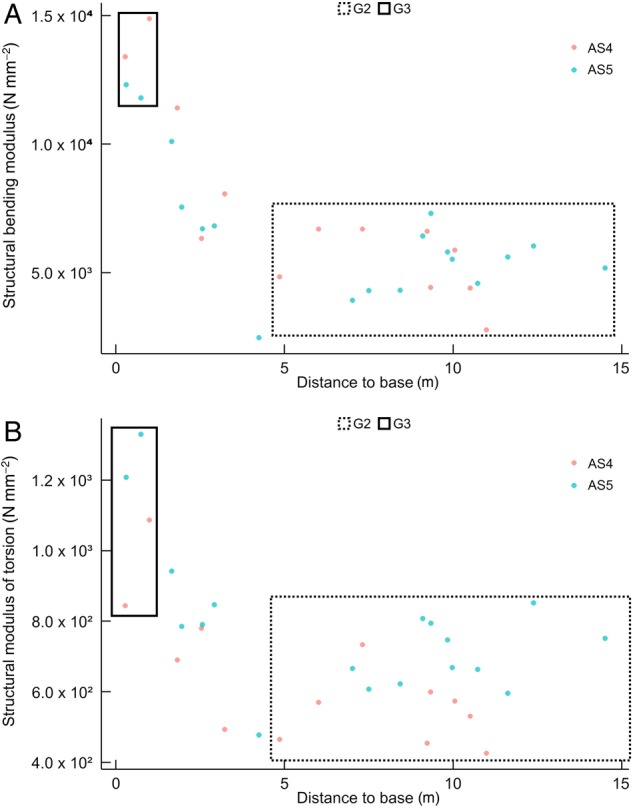
Changes of material properties, structural bending modulus (*E*) and structural modulus of torsion (*G*), along the stems of two aerial shoots (AS 4 and AS 5) of *F. indica*. Samples, which are assigned to the developmental stages G2 and G3, are highlighted by boxes with dashed or continuous lines. Due to spatial limitations in the greenhouse, the apical stages were cut back and, thus, cannot be plotted here. Old G3 segments close to the base of the shoots have highest material properties (A, structural bending modulus; B, structural modulus of torsion). Both *E* and *G* decrease with distance from the base and level off when the developmental stage G2 is reached.

Apical stem regions are followed by stem segments with almost constant material properties. The bending modulus of G2 samples was maintained between 4000 and 6000 N mm^−2^ (5192.7 ± 1308.1 N mm^−2^) (Figs 4A, 5 and 6A), while the modulus of torsion always lay between 400 and 800 N mm^−2^ (623.8 ± 121.0 N mm^−2^) (Figs 4A and 5 and 6B).

#### Stage G3

Old, basal stem segments have the highest material properties (Figs 4–6). The average bending modulus (11 673.9 ± 2030.1 N mm^−2^) is more than twice the average of that measured at the G2 stage. The average of the modulus of torsion (1074.6 ± 141.8 N mm^−2^) is almost twice as high as that of G2 segments (Fig. 5; Table 1).

**Table 1.** Biomechanical characteristics of developmental stages. Group G1 includes young, most apical stems (0–1 m from apex). G2 contains samples of 4–13 m distance to base. G3 consists of old, most basal stem fragments between 0 and 1 m distances to base. All moduli, as well as E/G and EI/GJ ratios, and diameter, are averaged and given with the corresponding standard deviation (mean ± SD).

Stage	*E* (N mm^−2^)	*G* (N mm^−2^)	*E*/*G*	*EI*/*GJ*	*d* (mm)	*n*
G1	1153.4 ± 991.1	155.6 ± 124.7	7.4 ± 4.1	3.5 ± 1.7	8.5 ± 1.0	6
G2	5192.7 ± 1308.1	623.8 ± 121.0	8.3 ± 2.2	4.2 ± 0.9	8.3 ± 1.6	19
G3	11 673.9 ± 2030.1	1074.6 ± 141.8	10.9 ± 2.6	5.5 ± 0.9	6.5 ± 1.1	9

Overall, the values of material properties decrease with increasing distance from the base (Fig. 5). The oldest and longest stems analysed (AS 4–5), with a maximum length of 15 m (Fig. 6), are characterized by a continuous decrease of moduli from the base (G3) towards the transition zone and reach almost constant moduli in the stage G2 (Fig. 6). Statistical analyses indicate a significant increase of the modulus of torsion and the bending modulus from G1 to G3 (*E*: χ^2^(6) = 226.16, *P*< 0.001; *G*: χ^2^(6) = 226.16, *P*< 0.001; Table 2). Although an increase of the *EI*/*GJ* ratio is indicated by the mean values of the developmental stages (Table 1), these differences are not highly significant in the pairwise Mann–Whitney *U*-test (*P*> 0.001; Table 2). This could be due to the low sample size per group. However, the increase of the *EI*/*GJ* ratio indicates an increase in anisotropy from G1 to G3 (Table 1). In general, all samples are easier to twist than to bend (*E*> *G*); thus, the ratio of *EI* and *GJ* is always above 1 (Fig. 4B and Table 1).

**Table 2.** Comparison of mechanical properties of the three developmental stages G1 (*n* = 6), G2 (*n* = 19) and G3 (*n* = 9). The Kruskal–Wallis test indicates significant differences between the groups (*E*: χ^2^(6) = 226.16, *P* < 0.001; *G*: χ^2^(6) = 226.16, *P* < 0.001; *EI*/*GJ*: χ^2^(6) = 226.16, *P* < 0.001). Significant differences in *E* and *G* for G1–G3 could be identified using the pairwise Mann–Whitney *U*-test (*post hoc*). Only G2 and G3 show differences in the *EI*/*GJ* ratio with *P* = 0.003.

	*E* (N mm^−2^)			*G* (N mm^−2^)			*EI*/*GJ*		
	G1	G2	G3	G1	G2	G3	G1	G2	G3
G1	–	<0.001	<0.001	–	<0.001	<0.001	–	0.400	0.289
G2	<0.001	–	<0.001	<0.001	–	<0.001	0.400	–	0.003
G3	<0.001	<0.001	–	<0.001	<0.001	–	0.289	0.003	–

While the plot of *E* against *G* shows distinct areas for all three developmental stages (Fig. 4A), the pattern in the plot of *EI* against *GJ* is not as clear (Fig. 4B). This is mainly due to differences of the stem diameter of the samples. Although the stem diameter of one aerial shoot is quite homogenous, the stem diameter between aerial shoots or between the main stem of an aerial shoot and its branches can differ greatly (Table 1). This has an influence on the geometrical factors, second moment of area (*I*) and torsional constant (*J*), which both contribute to flexural (*EI*) and torsional rigidity (*GJ*).

### Anatomy

**Figure 7.**
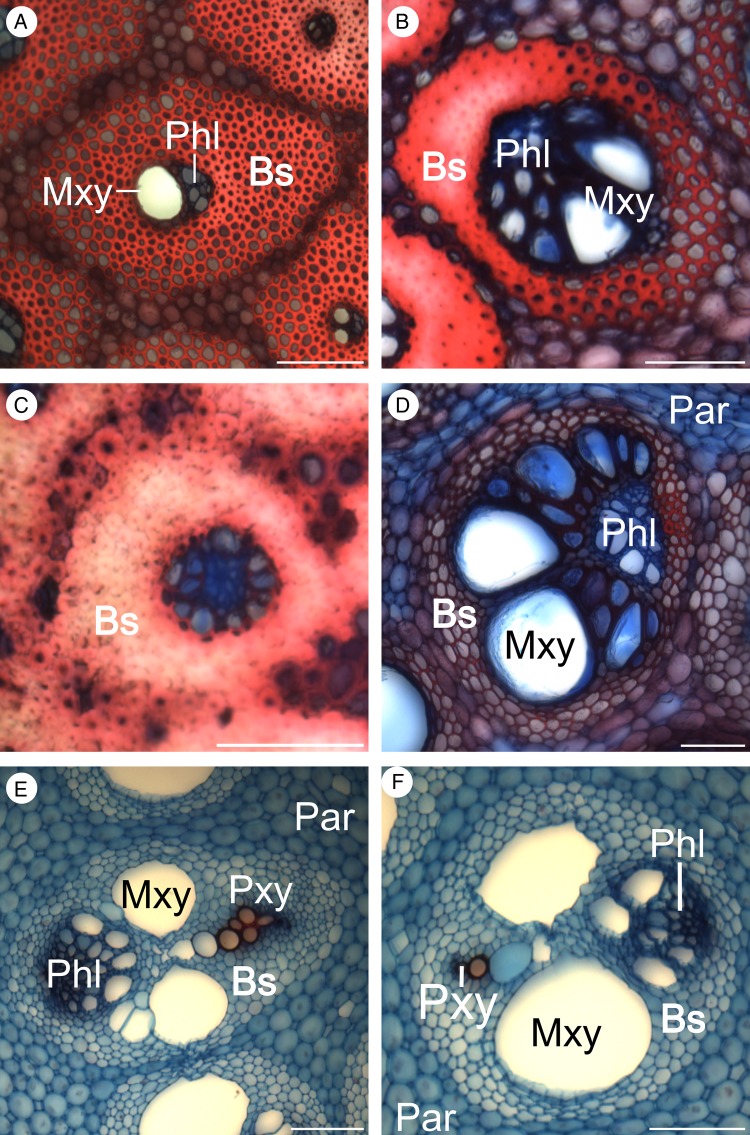
Variation of vascular bundles. (A and C) Vascular bundles of the peripheral region, with abundant and strongly lignified bundle sheaths, as well as reduced conductive tissues. (B and D) Vascular bundles at the transition zone of peripheral and central region. (B) shows higher bundle sheath density towards the periphery. (D) is characterized by a large number of conductive elements. (E and F) Vascular bundles of the central region of a young stem fragment differing in shapes from oval to circular, and having a large amount of non-lignified cells. Par, parenchyma; Phl, phloem; Pxy, protoxylem; Bs, bundle sheath; Mxy, metaxylem. Scale bars are 100 µm.

**Figure 8.**
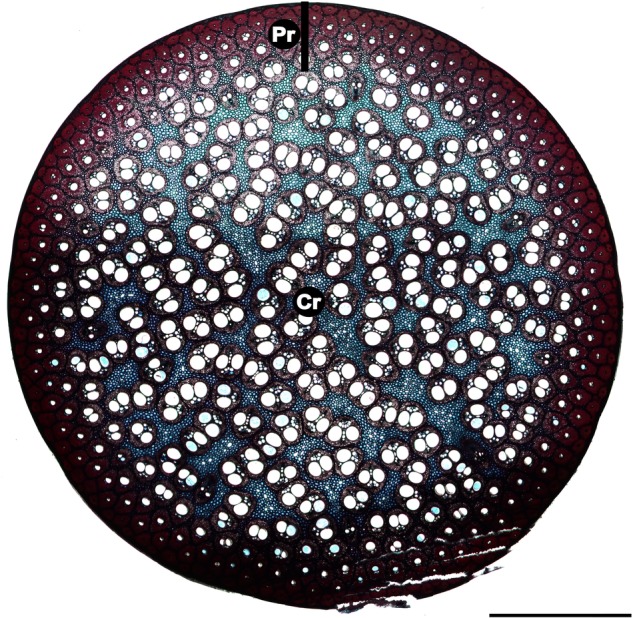
Transverse section of *F. indica*, without the leaf sheath. The stem is differentiated into two areas with specific anatomical characteristics. The region towards the periphery shows an increased number of vascular bundles and density of the bundle sheath. The central region shows a typical monocot stem anatomy, with scattered vascular bundles surrounded by parenchyma. Pr, peripheral region; Cr, central region. Scale bar is 2 mm.

**Figure 9.**
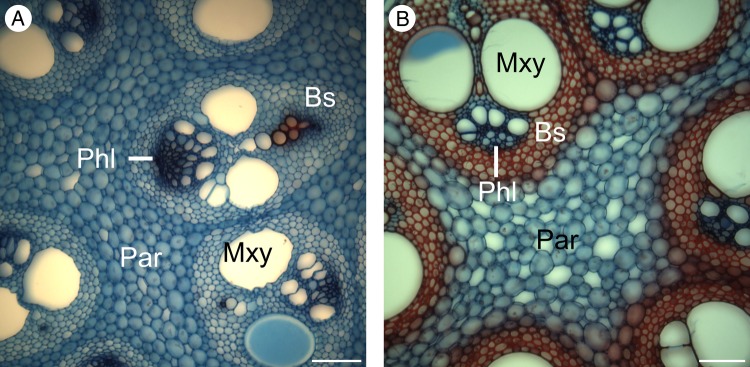
Transverse sections of two developmental stages from central stem regions of *F. indica*. Differences are not as pronounced as in the peripheral region. (A) Young apical stem sections show low degrees of lignification, with the exception of the protoxylem. (B) Differentiation processes of the central region involve lignification of the bundle sheath and the metaxylem. Par, parenchyma; Phl, phloem; Bs, bundle sheath; Mxy, metaxylem. Scale bars are 100 µm.

**Figure 10.**
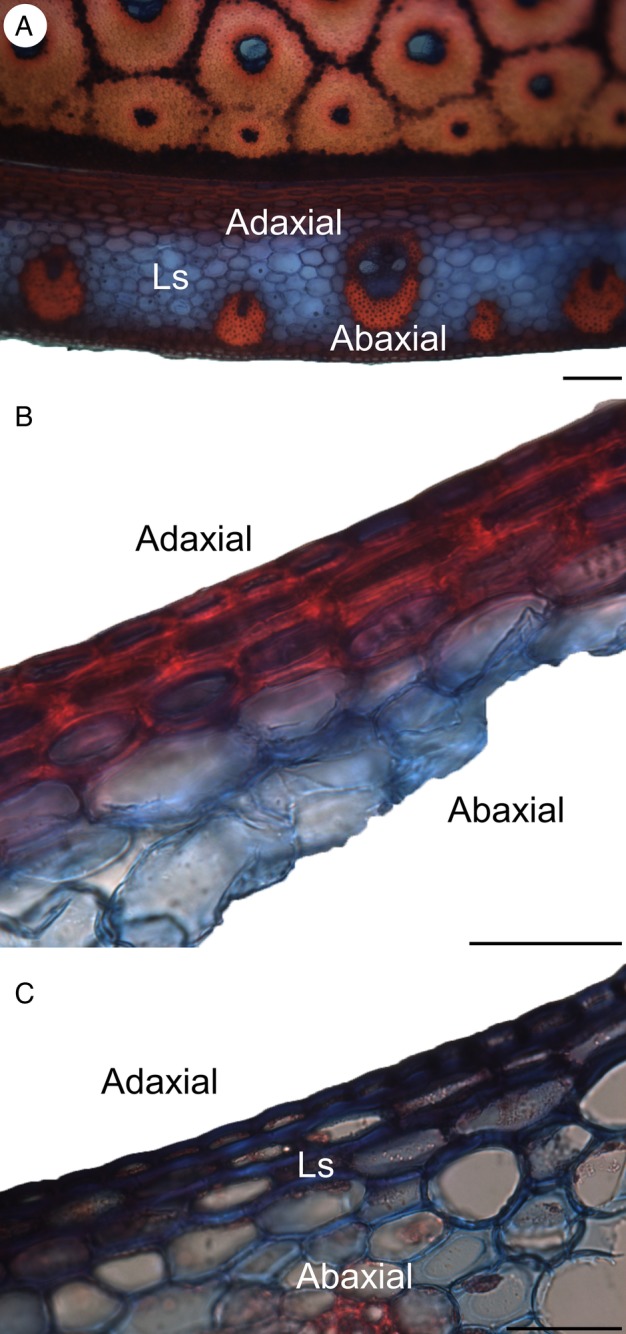
Developmental changes in the leaf sheath. (A) Close contact of leaf sheath and stem of developmental stage G2. (B) Lignification of leaf sheath tissue orientated towards the stem. (C) In younger stages, no lignification at the adaxial side of leaf sheaths is observed. Ls, leaf sheath. Scale bars are (A) 100 µm; (B and C) 50 µm.

**Figure 11.**
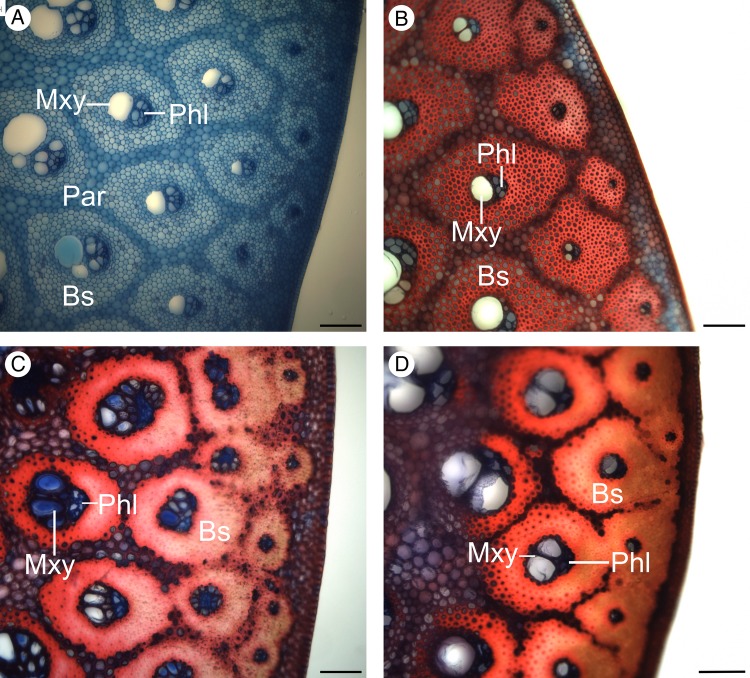
Details of developmental changes in transverse sections of the peripheral region of the stem, with increased maturity from A to D. (A) Young apical stem sections hardly show any lignification. Furthermore, vascular bundles are still separated by parenchyma. (B) Onset of lignification and increase of tissue density; bundle sheaths are still not fused. (C) Fusion of bundle sheaths and lignification of parenchyma increases. (D) In older, rigid and basal stems, maximal bundle sheath fusion and tissue density are observed. Par, parenchyma; Phl, phloem; Bs, bundle sheath; Mxy, metaxylem. Scale bars are 100 µm.

*Flagellaria indica* shows the characteristic monocot stem organization, with scattered vascular bundles surrounded by parenchyma. Each vascular bundle consists of phloem and xylem, and is protected by a sclerenchymatous bundle sheath. Vascular bundles vary in terms of vessel number and size, degree of lignification, thickness of cell walls and thickness of bundle sheath (Fig. 7). In general, the number of vascular bundles and the density of the bundle sheath increase towards the periphery of the stem. Thus, the stem is differentiated into two regions with specific anatomical characteristics, i.e. the peripheral and the central regions (Fig. 8). Conductive tissues of the vascular bundles in the peripheral region are reduced, while the amount of sclerenchymatous bundle sheath fibres is comparably high (Fig. 7A and B). In the central region, vessels are much larger and the bundle sheath fibres are less dominant (Figs 7E and F and 9). Whereas the parenchyma forms a distinct part of the central stem, it is reduced towards the periphery and/or lignified. Leaf sheaths closely envelope the stems (Figs 3 and 10A). The thin cortex of the stem consists of four to five cell layers outside the peripheral region of dense vascular bundles (Fig. 11A–C). In addition to these overall anatomical features, each developmental stage possesses particular anatomical characteristics.

#### Stage G1

In young apical stems, the peripheral regions are not yet or only slightly lignified (Fig. 11A and B), while vascular bundles of the central zone show lignification of protoxylem cells (Fig. 9A). The bundle sheaths of peripheral vascular bundles are clearly separated and the cell walls of fibre cells are thin.

#### Stage G2

**Figure 12.**
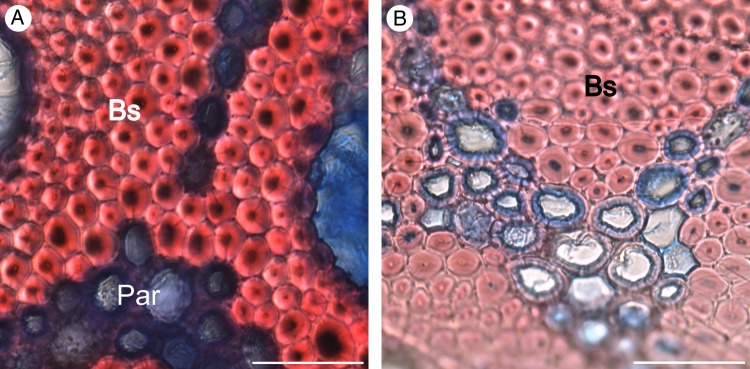
Fusion of bundle sheaths observed in the periphery of older stems (A) and lignification of parenchyma between bundle sheaths (B). Par, parenchyma; Bs, bundle sheath. Scale bars are 50 µm.

During further development, cell walls of the xylem and the bundle sheath of the central vascular bundles completely lignify while the parenchyma between the bundles remains largely non-lignified (Fig. 9). In the central region, no further distinct developmental differences between G1 and G2 were observed. However, in the peripheral region, tissue density and lignification increase during development (Fig. 11A–C), and cell walls of the fibres of peripheral bundle sheaths increase in thickness. Furthermore, the bundle sheaths of adjacent vascular bundles begin to fuse to a continuous layer of lignified cells in the peripheral region (Fig. 11C). This fusion is accomplished by lignification and cell wall thickening of parenchyma tissue between the vascular bundles (Fig. 12). The amount of fusion between adjacent vascular bundles is highest in the outer most ring of vascular bundles, which also shows the largest reduction of conductive tissue. Fusion affects three to four rings of vascular bundles. The pattern reveals a gradual decrease in the amount of fusion from periphery to centre (Fig. 11C).

#### Stage G3

**Figure 13.**
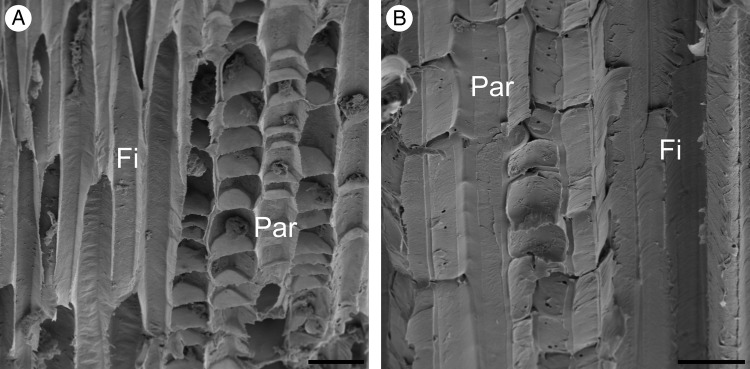
Longitudinal sections from the peripheral region of a stem of *F. indica*. (A) Scanning electron microscopic image of a young ontogenetic stage of the peripheral region. Between elongate fibre cells, small (∼12 µm) isodiametric cells of parenchyma are observed. (B) Scanning electron microscopic image of a mature stage from the peripheral region. Between fibrous cells, elongated (∼34 µm) parenchyma cells are visible. Par, parenchyma; Fi, fibres. Scale bars are 20 µm.

The largest amount of lignification and bundle fusion is reached in the most basal and rigid region of the stem and, therefore, increases with age. In addition, peripheral parenchyma cells show a notable elongation during maturity in addition to their lignification (Fig. 13). During development (from G1 to G3), the fibres of vascular bundle sheaths located between the peripheral and central region that are orientated towards the periphery increase in density while those directed towards the centre remain unchanged and are less prominently lignified (Fig. 11D). Changes in leaf sheath anatomy were also observed with respect to age-related lignification of the tissue orientated towards the stem (Fig. 10).

## Discussion

### Changes in growth habit, biomechanics and stem anatomy during ontogeny

Plant stems are fibrous composite materials and their mechanical traits result from mechanical properties of both fibre and matrix, as well as fibre orientation within the stem (Hornbogen *et al*. 2011). In *F. indica*, this is, without exception, reflected in a higher bending modulus compared with the modulus of torsion correspondingly leading to high values of *EI*/*GJ* (*EI*/*GJ* > 1; Table 1). A high *EI*/*GJ* ratio indicates a high degree of anisotropy, suggesting a strong lignification and degree of fibre orientation. In general, stems with a high proportion of parenchyma and, therefore, a high amount of hydrostatic tissue show a higher resistance to torsional loads (Niklas 1992; Vogel 1992).

Apical stem sections (G1) have the lowest moduli measured (Table 1; Figs 4 and 5). Lignification and cell wall thickening, both being of high mechanical relevance (Gierlinger and Schwanninger 2006; Horvath *et al*. 2010; Schreiber *et al*. 2010), have not yet been initiated, even though tissue distribution and longitudinal orientation of fibres in the stem is determined. Thus, the contribution of mechanically relevant tissues (i.e. with lignified and thickened cell walls) to the material properties is relatively low, Lignification and thereby stiffness of young stems increase with distance from the apex. Samples of G1, thus, include different degrees of lignification. However, their cell walls have not thickened and lignified as prominently as it is the case in older stages (G2 and G3) and bundle sheaths are not fused by lignified parenchyma cells in the peripheral region. A rapid increase of rigidity in apical parts of shoots is essential for the survival and ecology of climbing plants. It allows potential supports to be located and gaps in the vegetation to be successfully spanned until attachment is assured (Rowe *et al*. 2006). The *EI*/*GJ* ratio of G1 segments is low (Table 1, G1) and attributable to the large amount of parenchyma (Niklas 1992; Vogel 1992).

During development (G2), lignification and cell wall thickening of mechanical relevant tissues, and thus their contribution to the material stiffness, is increased. As a result, the *EI*/*GJ* ratio increases (Table 1). The fusion of bundle sheaths due to parenchyma lignification in the peripheral region increases the amount of sclerenchymatous tissue in the stem (Fig. 12), raising stiffness further compared with younger stages. The resulting gradient in tissue density towards the periphery is known to increase rigidity and to be congruent with the gradient of tension during bending and torsional loads (Amada *et al*. 1997). Tension increases with distance to the neutral axis or neutral plane and is highest in the most peripheral fibres (Bürgel 2005; Dankert and Dankert 2006; Läpple 2008; Wagner *et al*. 2012). Thus, the high density of mechanically relevant tissue in the periphery has a large influence on the material properties of the stem. G2 is characterized by somewhat consistent material properties (*E* and *G*) along most of the aerial stem. This mechanical architecture facilitates the climbing growth habit by reducing the risk of mechanical failure, e.g. allowing stem position to be maintained if attachment to supports is partially lost. This is especially beneficial in low height forest communities, such as mangroves or at forest margins, where climbers such as *F. indica* have no need to reach tree canopies of 50 m or more above the ground. These stiff stem characteristics, which are also found in Stage G3, are mainly brought about through changes in the peripheral region of the stem. These include larger numbers of vascular bundles and greater tissue density towards the periphery.

In basal stem segments (G3), bundle sheath fusion is highly advanced (Fig. 11). The vascular bundles of the outermost ring fuse completely, resulting in a cylinder of dense, stiff fibres and parenchyma cells. The latter are lignified and elongated (Fig. 13). Furthermore, most of the adjacent layer of vascular bundles fuses, although not to the same degree as in the outermost ring. Prominent fusion of bundle sheath, lignification and elongation of parenchyma cells increase the rigidity of basal stem regions by contributing to the material properties (*E* and *G*). The high *EI*/*GJ* ratio of old, basal stem segments (G3; Table 1) most likely depends on the degree of vascular bundle fusion in the stem periphery (Figs 11 and 12), resulting in a cylinder of stiff lignified fibre tissue with high resistance to mechanical loads. Furthermore, the elongation of parenchyma cells (Fig. 13) contributes to the direction dependency of mechanical properties and thereby to the anisotropy of the stem.

When taking the low safety factors of the long and slender shoots of *F. indica* into account (Niklas 1998; Niklas and Spatz 1999), it is evident that the amount of self-supporting growth must be limited. However, when assessing the energetic costs for developing stiffness in basal self-supporting stem regions (G3), it is apparent that it is energetically more beneficial to reduce tissue maturation processes in climbing stem regions (G2) because these are mechanically supported by hosts via the clinging leaf tendrils.

### Climbing habit of *F. indica* and other monocots

The general trend observed in *F. indica* broadly resembles that discovered for woody self-supporting trees and shrubs (Speck *et al*. 2003), with an overall decrease of stiffness from basal towards apical stem segments. In particular, the stems of Stage G2 reflect a semi-self-supporting stage with nearly constant material properties (Fig. 6). Thus, the biomechanical behaviour discovered for the climber *F. indica* differs from that observed for most climbers. Thus, while basal parts of the stem become more flexible with age in most climbers (e.g. Speck *et al*. 2003; Rowe *et al*. 2004; Wagner *et al*. 2014; Angyalossy *et al.* 2015), those of *F. indica* increase in stiffness reaching material properties comparable with those of the bamboo *Phyllostachys edulis* (Table 1). This is a tubular self-supporting member of the Poaceae with mean values of bending modulus ranging from 11 000 to 17 000 N mm^−2^ (Amada and Untao 2001). This demonstrates how little is known about climbing monocots outside of the palms and that further investigations of the growth habit of these plants are much needed. The few monocot climbers studied thus far suggest that the growth form diversity of monocots and in particular of monocot climbers is much higher than expected: *Desmoncus polyacanthos*, a climbing palm reaching stem diameters comparable with those of *F. indica*, possesses a less distinctive self-supporting phase and instead attaches rapidly to a support in dense vegetation, or discontinues apical growth by entering a latency period before growth continues (Isnard *et al*. 2005). The juvenile self-supporting phase of *Desmoncus orthacanthos* instead can, nevertheless, achieve an unsupported height of 2–3 m, but unlike in *F. indica*, this is effected via large basal stem diameter (up to 3 cm) and high stem stiffness, which changes little with distance from the base (Isnard *et al*. 2005).

In contrast to the climbing palms, which attach via cirri (modified leaf rachis) or flagella (modified inflorescences) (Isnard and Rowe 2008*b*; Isnard and Silk 2009; Rowe and Isnard 2009), *F. indica* climbs by means of leaf tendrils, which coil around mechanical hosts. While the diameter of potential supporting structures is limited, a strong and close contact to various kinds of support is achieved through tendrils thicken adaxially (S. Wagner, A. Rjosk and C. Neinhuis, unpubl. data). In contrast, the climber-support-connection of flagellate or cirri climbers like *Desmoncus* and *Calamus* is relatively loose and detachable (Isnard and Rowe 2008*b*; Rowe and Isnard 2009). While the latter can easily find new supporting structures after detachment, one main challenge for tendril climbers is the tight anchorage to the mechanical host, which can sway in the wind, expand or die back. Preliminary results have shown that the attachment of leaf tendrils of *F. indica* is relatively strong, facilitated by a high degree of lignification in the tendril and by friction between tendril and support (S. Wagner, A. Rjosk and C. Neinhuis, unpubl. data). If the stress acting on the tendril reaches a critical level, the tendril fails and separates the stem of *F. indica* from the host (S. Wagner, A. Rjosk and C. Neinhuis, unpubl. data). Thus, the integrity of the stem is preserved, and further attachment will occur via remaining leaf tendrils. The integrity of the stem is especially crucial for the survival of monocot climbers since damaged tissue, especially of the conducting vessels, cannot be repaired by cambial activity (Tomlinson and Zimmermann 2003).

### Climbing habit to escape constraints by primary growth

The climbing habit evolved several times independently within monocotyledons (e.g. *Bowiea volubilis*, *Bambusa moreheadiana*, *Calamus*, *Desmoncus* and *Flagellaria*). *Calamus* is assumed to be one of the most successful climbing genera and moreover comprises the longest known, singly rooted vascular plant species (Weinstock 1983; Uhl and Dransfield 1987; Isnard and Rowe 2008*a*). Since growth form diversity reaches its peak in the tropics, the respective climatic conditions might be a driving force towards the development of the climbing habit (Couvreur *et al*. 2014). In agreement, rattan palms as well as *Flagellaria* are distributed in the tropics and subtropics of the Old World (Weinstock 1983; Kubitzki *et al*. 1998; Rabenantoandro *et al*. 2007; Isnard and Rowe 2008*a*; Couvreur *et al*. 2014). Because climbing palms and *Flagellaria* are constitutively confined to primary growth and therefore unable to develop large diameter stems and height in order to escape shading by rainforest understorey, they have seemingly evolved alternative mechanical characteristics to survive the competition. These include specialized attachment structures, such as spiny structures of some climbing palms (Couvreur *et al*. 2014) or the tendrils of *F. indica*. Such attributes may have facilitated the evolution of several climbing monocot taxa. Climbing by means of a compliant stem is a limiting factor due to the anatomical constraints of monocot plants lacking secondary growth. Nevertheless, some monocot climbers, i.e. *Calamus*, have achieved high stem flexibility by loosing of the leaf sheath (Isnard and Rowe 2008*a*). The stem organization of monocots seems simple, but is, nevertheless, capable of developing contrasting growth forms by relatively simple modifications (Couvreur *et al*. 2014).

Although there are many monocot climbers, only a few species belong to the species-rich Poales (e.g. Tomlinson and Fisher 2000). The hypothesis that simple changes lead to a climbing habit in monocots is supported by the atypical climbing architecture of *F. indica*, which does not show the characteristic stem biomechanical pattern observed in non-monocot climbers. The modification towards the climbing habit in monocots might involve the development of attachment structures with appropriate mechanical characteristics of stems and leaf tendrils described here.

## Conclusions

Despite being confined by primary growth, monocot climbers can develop into large-bodied plants. In *F. indica*, maturation processes lead to higher densities of mechanically relevant tissues in the periphery of the stem. Attachment via leaf tendrils enables the transition from self-supporting to climbing growth followed by further substantial growth height thereby gaining access to high light intensities. Furthermore, adaptations to altered mechanical requirements, such as collapse of or disconnection from the mechanical support, become possible. Even though the range of mechanical modification in *F. indica* is limited by the lack of secondary cambial growth, this has not prevented the development of specialized attachment structures. The appearance of such structures has probably underpinned the evolution of numerous large-bodied climbing monocot taxa.

## Sources of Funding

This study was funded by budget resources of the chair for botany, TU Dresden.

## Contributions by the Authors

L.H. conducted the experiments, gathered all data and wrote the first draft of the manuscript. C.N. initiated the study and contributed to the improvement of the manuscript. S.T.W. participated to the realization of the study, supported the experimental work and contributed to the improvement of the manuscript.

## Conflict of Interest Statement

None declared.
